# Evidence for intact stimulus-specific neural adaptation for visual objects in schizophrenia and bipolar disorder: An ERP study

**DOI:** 10.1371/journal.pone.0221409

**Published:** 2019-08-20

**Authors:** Jonathan K. Wynn, Stephen A. Engel, Junghee Lee, Eric A. Reavis, Michael F. Green

**Affiliations:** 1 Mental Illness Research, Education and Clinical Center, Veterans Affairs Greater Los Angeles Healthcare System, Los Angeles, California, United States of America; 2 Semel Institute for Neuroscience and Human Behavior, University of California, Los Angeles, California, United States of America; 3 Department of Psychology, University of Minnesota, Minneapolis, Minnesota, United States of America; National Autonomous University of Mexico, MEXICO

## Abstract

People with schizophrenia (SZ) or bipolar disorder (BD) experience dysfunction in visual processing. Dysfunctional neural tuning, in which neurons and neuronal populations are selectively activated by specific features of visual stimuli, may contribute to these deficits. Few studies have examined this possibility and there are inconsistent findings of tuning deficits in the literature. We utilized an event-related potential (ERP) paradigm to examine neural adaptation for visual objects, a measure of neural tuning whereby neurons respond less strongly to the repeated presentation of the same stimulus. Seventy-seven SZ, 53 BD, and 49 healthy comparison participants (HC) were examined. In three separate conditions, pictures of objects were presented repeatedly: the same object (SS), different objects from the same category (e.g., two different vases; SD), or different objects from different categories (e.g., a barrel and a clock, DD). Mass-univariate cluster-based permutation analyses identified electrodes and time-windows in which there were significant differences between the SS vs. DD and the SD vs. DD conditions. Mean ERP amplitudes were extracted from these clusters and analyzed for group differences. Results revealed a significant condition difference over parieto-occipital electrodes for the SS-DD comparison between 109–164 ms and for the SD-DD comparison between 78–203 ms, with larger amplitudes in the DD compared to either SS or SD condition. However, there were no significant differences in the pattern of results between groups. Thus, while we found neural adaptation effects using this ERP paradigm, we did not find evidence of group differences. Our results suggest that people with SZ or BD may not exhibit deficits in neural tuning for processing of visual objects using this EEG task with rapidly presented stimuli. However, the results are inconsistent with other studies using different methodologies (e.g., fMRI, behavioral tasks) that have found tuning deficits in people with schizophrenia.

## Introduction

People with schizophrenia (SZ) exhibit visual processing abnormalities [1–3], which have downstream effects on daily functioning [4, 5]. Other patient populations, such as bipolar disorder (BD), share phenotypic characteristics and risk factors with SZ [6–8], and also exhibit visual processing abnormalities [9, 10]. While the nature of these visual processing deficits is not fully understood, one possible underlying mechanism that has received recent attention is stimulus selectivity, which is related to neural tuning [11, 12].

Neurons and neuronal populations are selectively activated by, or “tuned to,” specific features of visual stimuli. One well-known example is of neurons in early visual cortex (i.e., V1) that are tuned to visual stimuli presented at a specific orientation; a given V1 neuron will respond most strongly to one particular orientation and gradually less strongly as the mismatch between the preferred orientation and stimulus increases [13].

Non-invasive measures of neural activity, including functional magnetic resonance imaging (fMRI), magnetoencephalography, and event-related potentials (ERPs), can also provide indirect measures of neural tuning [14–17]. In particular, a phenomenon known as repetition suppression or stimulus-specific neural adaptation has been used to infer tuning specificity. Repetition suppression occurs when the same neurons are activated multiple times in rapid succession, such as when the same stimulus is presented repeatedly. When different stimuli are presented in alternation, repetition suppression can also occur to the extent that the two stimuli share visual features that neurons in the responding population are tuned to [18–20]. For example, if two stimuli with the same orientation features but different colors were presented in rapid succession, repetition suppression effects would be expected among neurons tuned to orientation but not those tuned to color. Specificity of neural tuning in particular visual feature domains can be estimated by presenting stimuli with varying degrees of similarity in rapid alternation and measuring repetition suppression effects. If neurons that respond are finely tuned to that featural domain, different neural populations should respond to the two stimuli and little repetition should occur, but if responding neurons are broadly tuned to that featural domain, many of the same neurons should respond to both stimuli and repetition suppression should be evident.

Object processing is a key domain of visual perception in which the specificity of neural tuning can be inferred using stimulus-specific neural adaption. The lateral occipital cortex (LOC) is a region that responds preferentially to visual objects rather than other visual stimuli (e.g., simple patterns, scrambled images, etc.) [21–23]. Deficits in object processing have been shown behaviorally, with event related potentials (ERPs), and with functional magnetic resonance imaging (fMRI). Behaviorally, people with schizophrenia often have difficulties identifying or detecting objects. These deficits have been demonstrated using visual backward masking tasks (e.g.,[24, 25]) and with tasks requiring subjects to identify objects that have been fragmented (i.e., pieces of the picture are missing, making it difficult to recognize the object; [26]). It has also been shown that behavioral measures of perceptual closure (i.e., object recognition) are correlated with psychotic symptoms as assessed with the Positive and Negative Syndrome Scale (PANSS) [27].

Further evidence comes from ERP studies. It has been reported that people with schizophrenia have reduced amplitudes of the perceptual closure negativity (N_cl_) [28], an ERP seen over parieto-occipital regions with generators located in LOC [29]. The N_cl_ is increased in amplitude when an object is easily recognized versus when the object is occluded in some manner (in this case, presenting fragments of the object). Amplitude reductions of the N_cl_ are also correlated with higher scores on the total, positive, and general symptom scales of the PANSS [26]. Functional MRI studies have also shown that people with schizophrenia have functional deficits in object processing in LOC, with findings of reduced activation [26, 30, 31], abnormal functional connectivity [32], as well as more diffuse activation (i.e., increased extent of activation) across the cortex [33]. Furthermore, people with schizophrenia exhibit structural abnormalities, with findings of reduced cortical thickness in LOC compared to healthy comparison participants [34]. Taken together, people with schizophrenia have behavioral and neural deficits when processing visual objects, which in turn contribute to the symptom severity.

However, evidence for *tuning deficits* in object processing in SZ or BD in LOC is mixed. Like other visual areas, LOC contains neuronal populations that are tuned to and respond selectively to particular object characteristics [35, 36]. Our own group has shown that people with SZ and BD exhibit tuning deficits in LOC using a fMRI adaptation paradigm [37], while a different study using multivariate pattern analysis of object categorization did not reveal any tuning deficits [38]. One potential reason for these discrepant results is the low temporal resolution of fMRI. ERPs, with their high temporal resolution, may be better suited to resolving short-lived repetition suppression effects that are missed due to the long timeframes (hundreds to thousands of milliseconds) examined in fMRI.

In the current study we examined stimulus-specific neural adaptation for objects using a rapid serial visual presentation ERP paradigm in SZ, BD and a healthy comparison (HC) sample. Three separate conditions were presented in 90 s blocks: the same object repeated (SS), different objects from the same category repeated (SD), and different objects from different categories repeated (DD). We hypothesized that neural responses would be lowest for SS, highest for DD, and intermediate for SD conditions. We further hypothesized that SZ would show less neural adaptation compared to HC because of reduced neural tuning for object features in SZ, with BD showing response patterns intermediate to SZ and HC.

## Materials and methods

### Participants

Participants came from a National Institutes of Mental Health-sponsored study of visual processing in major mental illness (“Visual Tuning and Performance in Schizophrenia and Bipolar Disorder”, MH095878, PI: Michael Green). Many of the participants in the current study participated in other published studies resulting from this project (e.g., [20, 38]). In total, 77 people with schizophrenia (SZ), 53 people with bipolar disorder (BD), and 49 healthy control participants (HC) participated in this electroencephalography (EEG) study. All participant eligibility criteria and methods to assess clinical diagnosis and symptoms have been previously published in detail ([20, 38]).

All patient participants were clinically stable outpatients with a Diagnostic and Statistical Manual of Mental Disorders– 4^th^ Edition (DSM-IV) diagnosis of either schizophrenia or bipolar disorder, recruited from the VA Greater Los Angeles Healthcare System (VAGLAHS) and outpatient board and care facilities and clinics in the greater Los Angeles area. All patients were receiving clinically determined doses of medication and tested outside of mood episodes. Healthy participants were recruited using ads placed on the internet (e.g., Craigslist). All procedures were approved by the Institutional Review Boards of VAGLAHS and the University of California, Los Angeles, with participants providing written informed consent. All participants were assessed for their capacity to provide informed consent using the IRB-approved Mental Illness Research, Education and Clinical Center (MIRECC) Evaluation of Capacity to Sign Consent form, which assesses a participant’s level of understanding of the nature of the study, the potential risks and benefits, and what their rights as a research participant are.

Inclusion criteria for all participants were: a) age 18–65, b) understand English sufficiently to understand the procedures and interviews, c) IQ > 70 and no developmental disability based on chart review, d) no history of neurological disease (e.g., epilepsy), e) no evidence of past serious head injury loss of consciousness > 1 hour, neuropsychological sequelae, or cognitive rehabilitation post-head-injury, f) no sedatives or benzodiazepines 12 hours prior to testing, g) normal or corrected vision, h) no positive urine toxicology screening at the time of assessment, i) no substance or alcohol dependence three months prior to participation or substance or alcohol abuse one month prior to participation, and j) no mood episode meeting clinical criteria for depression, mania or hypomania in the past two months.

Inclusion criteria for patient participants only included: a) diagnosis of schizophrenia or bipolar disorder based on the Structured Clinical Interview for DSM-IV Axis I Disorders (SCID-I) (First et al., 1997), and b) clinical stability (i.e., no inpatient hospitalizations during past 3 months, no psychoactive medication changes 4 weeks prior to enrollment, including change in medication type or dosage). Inclusion criteria for healthy controls only included: a) no history of psychotic disorder, bipolar spectrum disorder, or other major mood disorder based on SCID-I interview (First et al., 1997) or of a personality disorder in the schizophrenia spectrum (including avoidant, paranoid, schizotypal, or schizoid) or borderline personality disorder based on the Structured Clinical Interview for DSM-IV Axis II Disorders (SCID-II) (First et al., 1996), and b) no psychotic or bipolar disorder in first-degree relatives, based on participant report.

All clinical interviewers were trained through the Treatment Unit of the Department of Veterans Affairs Desert Pacific Mental Illness Research, Education, and Clinical Center, and met a minimum kappa of 0.75 for psychotic and mood items (Ventura et al., 1998). To corroborate self-report information when necessary, patients’ medical records and reports from their clinicians were examined if available. Clinical symptoms for the patient groups were assessed using the Brief Psychiatric Rating Scale (BPRS), Young Mania Rating Scale (YMRS), and Hamilton Depression rating scale (HAMD) (Hamilton, 1960; Ventura et al., 1993; Young et al., 1978).

### Task

**Fig 1:**
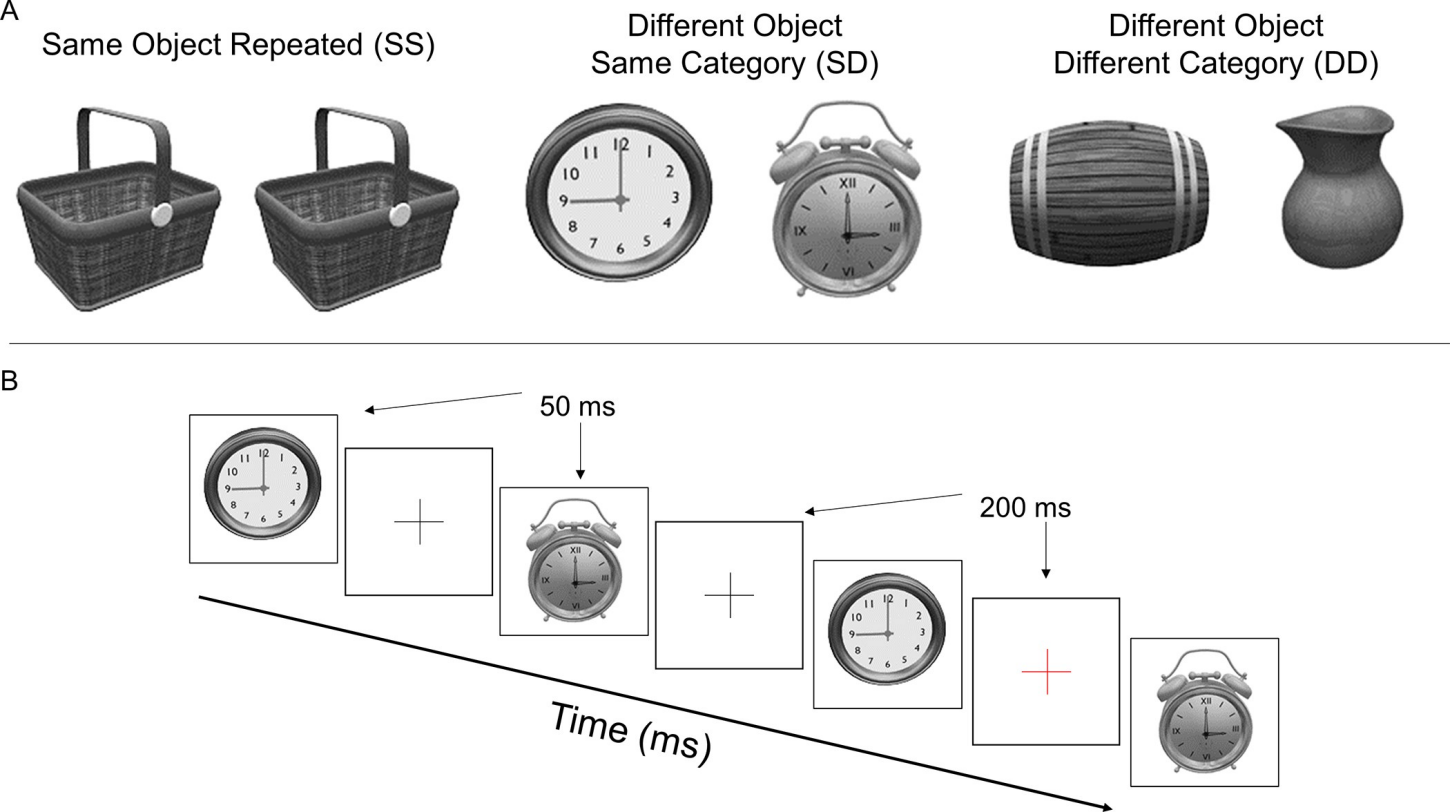
**A)** Examples of stimuli used in the three separate conditions (SS, SD, DD). **B)** Schematic diagram of sequence of presentation. Visual objects were presented for 50 ms with a 200 ms interstimulus interval (ISI). Fixation crosses were present during the ISI. As catch trials, the color of the fixation cross would occasionally change red to which the participant needed to make a manual response.

Visual stimuli were drawn from the Object Databank database of visual objects (www.tarrlab.org). Stimuli were shown in 3 separate, counterbalanced blocks. There were three possible conditions: the same object repeated (SS) such as the same picnic basket repeated; 2 different objects from the same category alternately repeated (SD) such as two different clocks; and 2 different objects from different categories alternately repeated (DD) such as a barrel and a vase (see Fig 1A for examples of stimuli). Three different sets of stimuli were utilized, each containing 5 objects. The specific objects were randomized across conditions, and order of conditions was randomized across subjects. Stimuli ranged in size from 120 x 120 pixels to 180 x 180 pixels, changing randomly throughout each block so the objects had slightly different retinal locations from trial to trial to limit local adaptation effects.

Stimuli were presented at fixation on a liquid crystal display monitor set at 1920 x 1080 pixels, running at 120 Hz. The experiment was programmed in E-Prime 2.0 (Psychology Software Tools, Inc., Sharpsburg, PA). Participants sat 1 m away from the screen. Stimuli were presented in a rapid serial visual presentation format every 250 ms (50 ms stimulus duration, 200 ms interstimulus interval [ISI]). Each block consisted of a single condition and lasted a total of 90 s, with a total of 360 stimuli shown in each block (see Fig 1B for an example segment of a block). During the ISI a central fixation cross was presented. For most of the ISIs, the fixation cross was black; however, occasionally (12 times per block) the color would change to red. As a validity check to ensure that participants’ eyes were open and fixated on the center of the screen, participants were instructed to press a mouse button whenever they saw the red fixation cross. Performance in all groups was above 87% for all three conditions, with no group or condition effects on performance.

### EEG acquisition and analysis

EEG was recorded using a BioSemi ActiveTwo system (BioSemi B.V., Amsterdam, Netherlands) with sintered Ag/AgCl active electrodes and 64 channel electrode caps. Two electrodes, placed above and below the left eye, were used to measure vertical electrooculography, and two electrodes, placed at the outer canthus of each eye, were used to measure horizontal electrooculography. Additional electrodes were placed on the left and right mastoid, and on the tip of the nose. Each electrode was measured online with respect to a common mode sense electrode during data collection, forming a monopolar channel. Data were re-referenced offline to the grand average of all scalp electrodes. Continuously recorded EEG was digitized at 1024 Hz and subsequently processed offline. Eye-blink artifacts were corrected offline using established methods [39].

We analyzed time-locked ERPs to all stimuli in each condition block. Stimuli were segmented into epochs of -50 to +250 ms relative to the onset of the stimulus, filtered between 1–20 Hz (24 dB/oct rolloff), baseline corrected using the -50 to 0 ms baseline prestimulus period, and subjected to artifact rejection of +/- 75 μV over all 66 scalp electrodes. Remaining trials were then averaged to derive ERP waveforms for each of the three conditions. There was a median of 360 trials accepted per condition per group, with no significant differences between conditions or groups.

We conducted data-driven cluster-based permutation analyses of difference waves [40, 41] to determine which electrodes and time windows showed significant differences between the conditions. We examined difference waves for the following comparisons: SS-DD, SD-DD, and SS-SD. For the cluster-based permutation tests, we used a cluster mass statistic [42] with a family-wise alpha level of 0.05. This method identifies and clusters together and sums results from neighboring time points and electrodes based on their significance and proximity. In an analysis involving all participants (irrespective of diagnosis), difference wave values at each time point and electrode were subjected to one-sample t-tests (null hypothesis: mean = 0). A significant (p < 0.05) t-value was then assigned to a particular cluster if it was temporally adjacent to another time point or spatially adjacent to a neighboring electrode that also had a significant t-value. Adjacent electrodes had to be within approximately 5.44 cm of one another to be considered neighbors. As recommended [40, 43] and used in prior studies [44], data were down-sampled to 128 Hz, and all time points between 50 and 200 ms (21 time points) at 20 parieto-occipital scalp electrodes (P1, P2, P3, P4, P5, P6, P7, P8, P9, P10, PO3, PO4, PO7, PO8, O1, O2, Pz, POz, Oz, Iz) were included (i.e., 420 total comparisons). These constraints (limiting data samples and electrodes) increase power to detect real differences in relevant time windows and electrodes. T-tests were performed for each comparison using the original data and 5,000 random permutations. For each permutation, all t-scores corresponding to uncorrected p-values of 0.05 or less were formed into clusters. The t-scores in each cluster were summed, and absolute value of the most extreme scores in each of the 5,000 sets of tests was recorded and used to estimate both sides of the cluster distribution of the null hypothesis, which was assumed to be symmetric [42]. Any cluster in the original data set that exceeded critical values in either tail of this null distribution was considered statistically significant. The results are depicted as a raster diagram highlighting in color the time points and electrodes belonging to a significant cluster.

If significant clusters were identified, we then extracted the mean amplitude of activity averaged over the specific time window and electrodes identified. We then examined: a) if there were any significant group differences in the differences between conditions, and b) how the wave amplitude changes over the course of the block (i.e., a time effect). We analyzed the data with a repeated measures analyses of variance (rmANOVA) with group (SZ, BD, HC) as a between-subjects factor and condition (SS, SD, DD) as a within-subjects factor. To examine the time effect, activity was averaged in 10 s time blocks. Visual inspection of pilot data indicated very minor changes in amplitude after the first 70 sec. We therefore did not analyze the last 20 s of data, and only examined change in activity over the first 7 time blocks. An additional rmANOVA was run which now included time (7 time blocks) as a within subjects factor.

## Results

The final anonymized data set necessary to replicate our study findings can be found on the Open Science Framework (https://osf.io/ks5a9/). Demographics and symptom ratings appear in Table 1. Groups were well matched on key demographics, including age, gender, and parental education, though they did differ significantly on personal education as expected. Sixty-seven SZ were on a second-generation antipsychotic, and 10 either were not taking or did not report taking an antipsychotic. Twenty-eight BD were on a second-generation antipsychotic. Chlorpromazine (CPZ) equivalent doses were calculated [45] for those with a known dosage and are shown in Table 1.

**Table 1:** Demographics and clinical characteristics.

	Schizophrenia (n = 77)	Bipolar Disorder (n = 53)	Healthy Control (n = 49)
Age	47.2 (12.7)	44.7 (12.2)	48.5 (8.1)
Gender (F:M)	26:51	25:28	24:25
Education	12.6 (1.8)^a^	14.6 (2.5)	14.4 (1.7)
Parental Education	13.7 (3.3)	14.8 (3.4)	14.5 (3.2)
Ethnicity Hispanic:Non-Hispanic	11:66	11:41	8:41
Race Black:White:Other^+^	29:43:5	10:33:8	12:26:11
Duration of Illness (Years)	25.4 (13.2)	25.5 (15.0)	
CPZ Equivalent (mg)	118.5 (188.5)	118.7 (176.1)	
RFS			
Work^b^	2.8 (1.9)	4.3 (2.0)	
Independent Living^b^	4.7 (1.4)	6.1 (1.1)	
Family^b^	5.1 (1.9)	5.9 (1.4)	
Social^b^	4.3 (1.8)	5.6 (1.5)	
MCCB Composite^c^	34.8 (12.5)	42.9 (11.7)	47.0 (11.2)
Symptoms			
BPRS Positive^b^	1.9 (0.9)	1.1 (0.3)	
CAINS MAP^b^	14.7 (6.6)	10.5 (5.8)	
HAM-D	6.5 (5.0)	6.5 (4.9)	
YMRS^b^	4.2 (3.7)	3.4 (4.4)	

a = SZ < BD, HC, p < 0.05 Bonferroni-corrected

b = SZ < BD, p < 0.01

c = SZ < BD, HC, p < 0.01; BD = HC

^+^Note: some chose not to disclose their race

CPZ = chlorpromazine equivalent; RFS = Role Functioning Scale; MCCB = MATRICS Consensus Cognitive Battery; BPRS = Brief Psychiatric Rating Scale; CAINS MAP = Clinical Assessment Interview for Negative Symptoms—Motivation and Pleasure subscale; HAM-D = Hamilton Depression Rating Scale; YMRS = Young Mania Rating Scale

**Fig 2:**
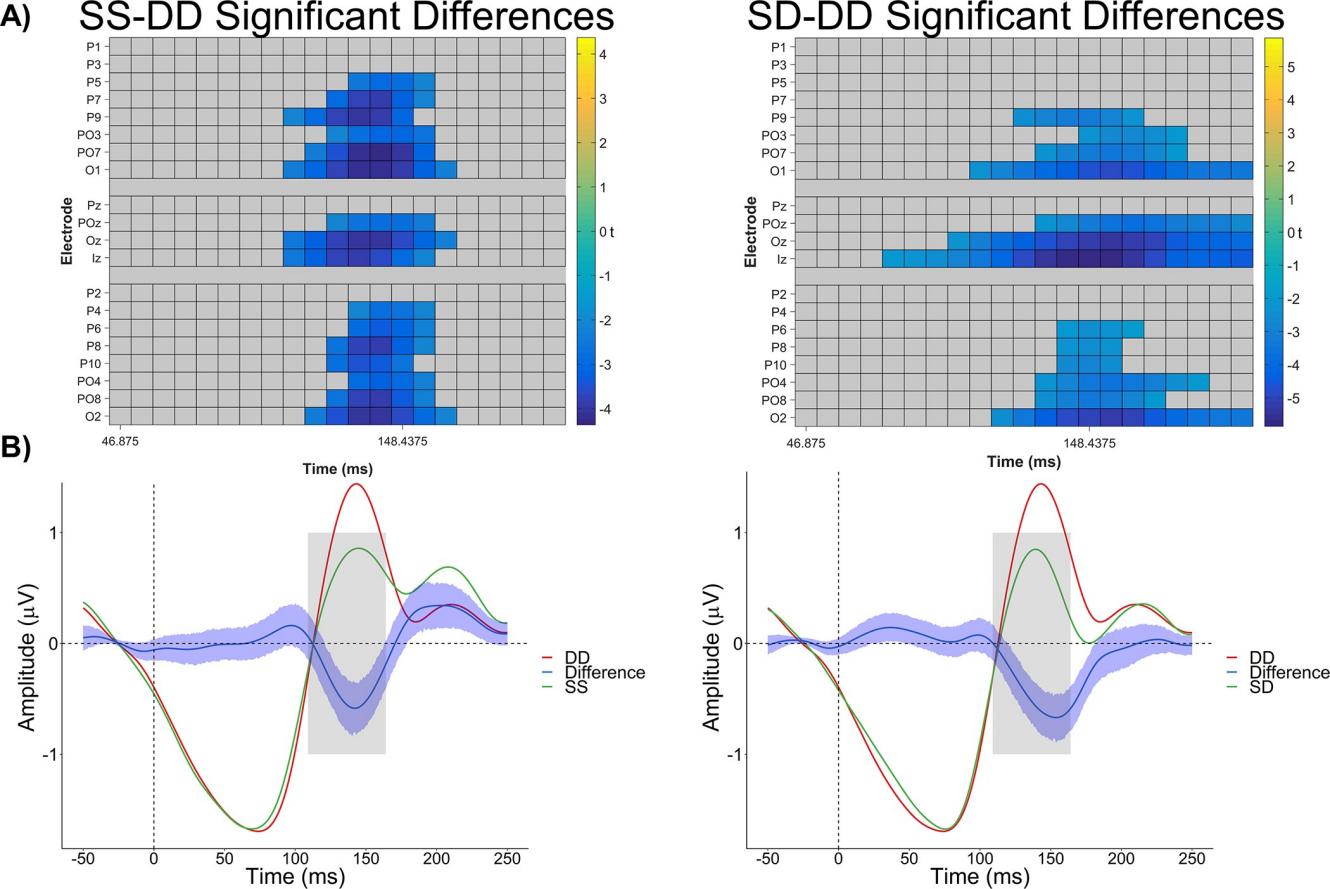
**A)** Raster diagram of significant between condition effects for the SS-DD (left) and SD-DD (right) comparisons, averaged across groups. Blue colored squares indicate significantly lower amplitudes compared to the DD condition. **B)** Event-related potential waveforms and between-condition difference wave, averaged across groups. Color shaded area surrounding the waveform indicated 95% confidence interval. Grey colored box indicates time window (109–164 ms) analyzed for potential group effects.

**Fig 3:**
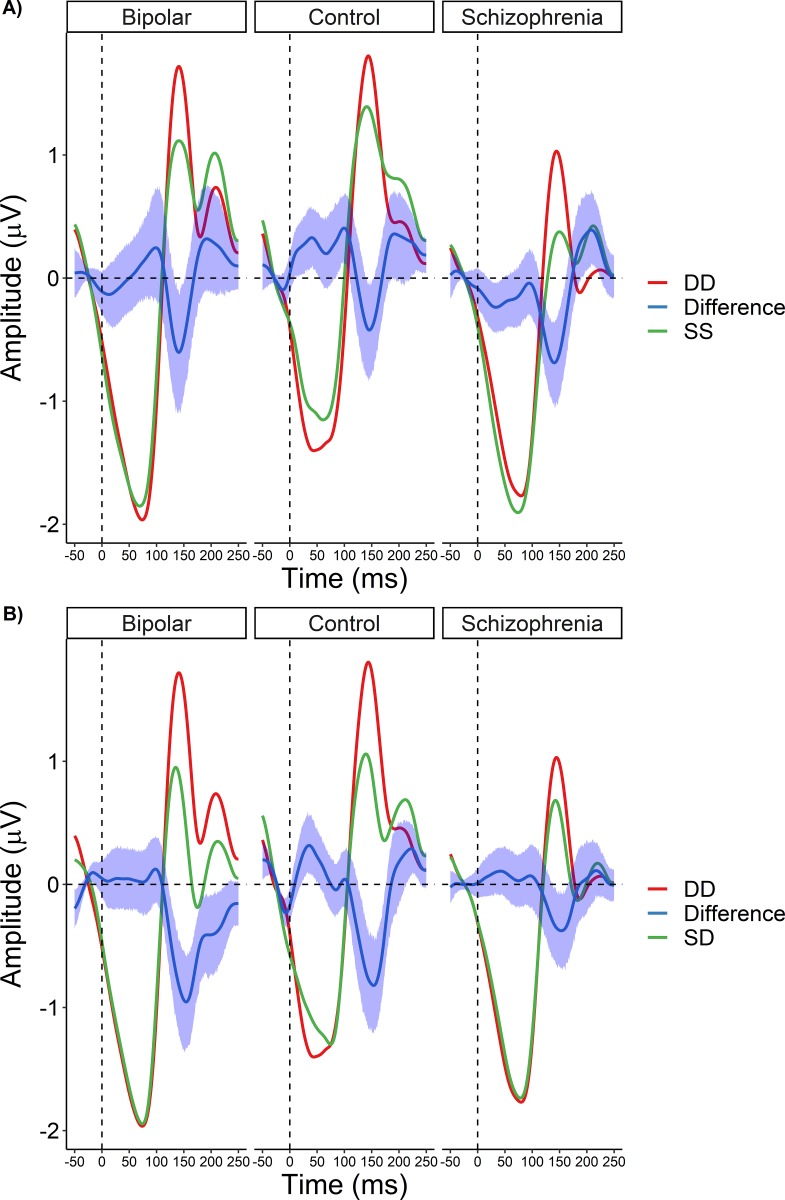
**A)** Event-related potential waveforms and between-condition difference wave for the SS-DD comparison, plotted separately for each group. Color shaded area surrounding the waveform indicates 95% confidence interval. **B)** Event-related potential waveforms and between-condition difference wave for the SD-DD comparison, plotted separately for each group. Color shaded area surrounding the waveform indicates 95% confidence interval.

The raster plots for the mass univariate analyses and the ERPs for each condition averaged across the three groups are shown in Fig 2 and Fig 3 shows waveforms and difference waves separately for each group. For the SS-DD comparison, a cluster was found showing significantly lower amplitudes in the SS compared to the DD condition between 109–164 ms across most of the electrodes that were examined. For the SD-DD comparison, a similar cluster was found showing significantly lower amplitudes in the SD compared to the DD condition. The time window was slightly more broadly distributed (78–203 ms) and included fewer electrodes. There were no significant clusters for the SS-SD difference wave.

**Fig 4:**
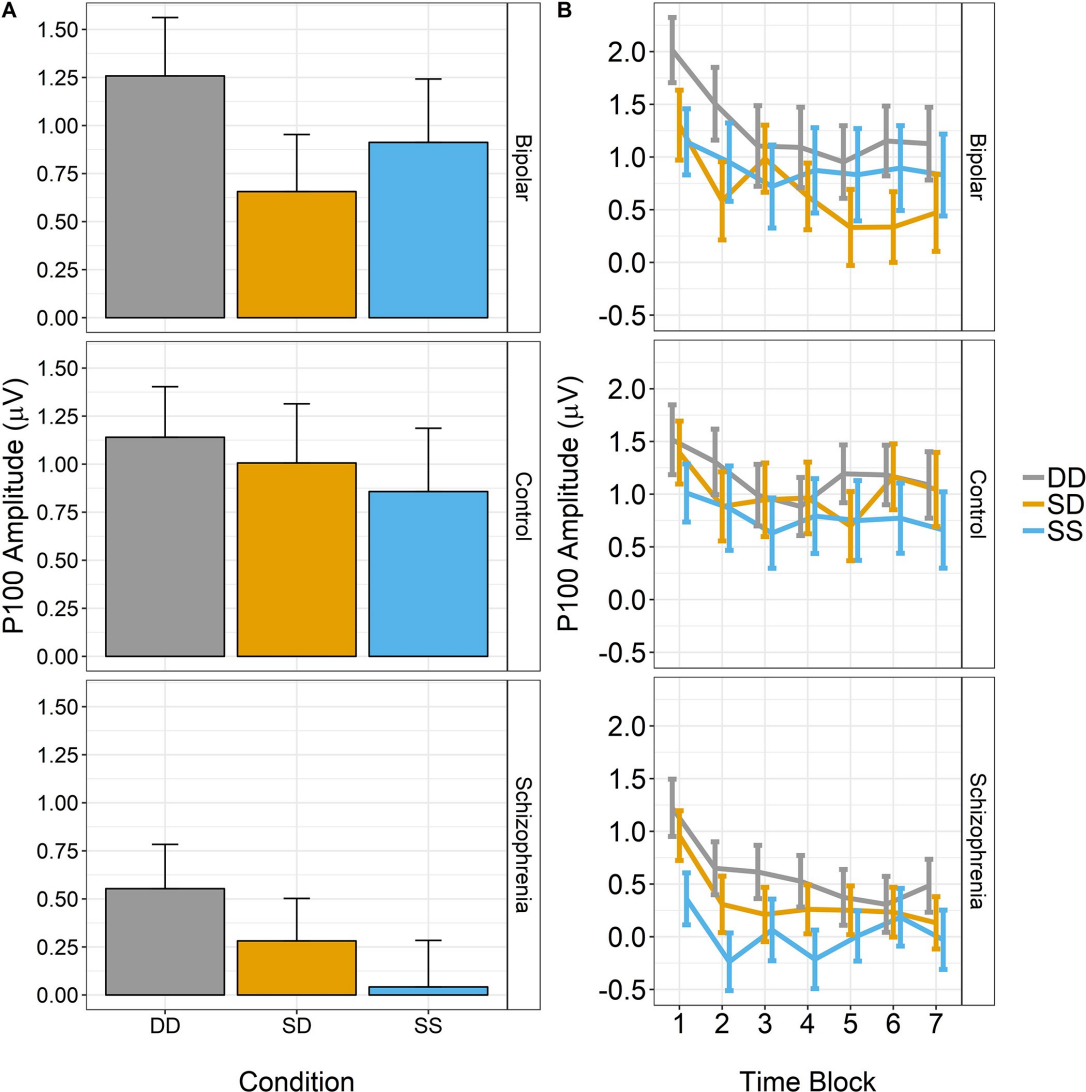
**A)** Mean amplitude (+/- 1 standard error) for each of the three conditions, collapsed across group. DD had significantly higher amplitude compared to SS and SD. **B)** Mean amplitude (+/- 1 standard error) for each of the three conditions as a function of time in 10 s blocks (X-axis), collapsed across group. All three conditions exhibited significant reductions in amplitude over the course of 70 seconds of stimulus presentation.

As there was considerable overlap in the clusters, we extracted the activity from the time window and electrodes identified in the SS-DD condition for follow-up analyses. A single measure of activity for each of the three conditions was computed by averaging over all electrodes showing significant differences within the 109–164 ms time window and subsequently used in the rmANOVAs. Using these values, we next examined if there were group differences in the pattern of activation across the conditions. Results of the rmANOVA revealed a significant main effect of condition (F_2,352_ = 6.12 p < 0.002), as expected given the results of the mass univariate analysis; however, there was no significant main effect of group nor a significant group X condition interaction. Follow-up Bonferroni-corrected contrasts on the condition main effect revealed that amplitudes were significantly lower for the SS and SD conditions compared to DD, p’s < 0.003 (Fig 4); there was no difference in activation between the SS and SD conditions.

We next analyzed how the amplitude of the ERP changed over the course of the block. Results revealed significant main effects of condition (F_2,352_ = 7.38, p < 0.001) and time (F_6,1056_ = 17.41, p < 0.001); the group main effect was not significant. Additionally, there was a significant condition X time interaction (F_12,2112_ = 2.12, p = 0.023); there were no other significant interactions. The effect of condition was due to significantly lower amplitudes in the SS and SD conditions compared to DD condition (p’s < 0.003). The significant time effect revealed that amplitudes decreased over time. The condition X time interaction was followed up by comparing subsequent time bins using paired t-tests (e.g., 10s vs. 20s, 20s vs. 30s, etc.), separately for each condition. These tests revealed that the decline over time was fastest in the SS and SD conditions (i.e., responses “bottomed-out” faster) compared to the DD conditions, with significant differences only seen for the 10s-20s comparison; the DD condition differences for the 10s-20s and 20s-30s condition.

## Discussion

We examined whether patients with SZ or BD exhibit stimulus-specific neural adaptation deficits for visual objects. We found that a rapid serial visual presentation paradigm to assess stimulus selectivity / neural tuning showed the expected pattern of results: stronger neural responses when object from different categories were presented in alternation, compared to weaker responses when the same object or two objects from the same category were presented under the same conditions. These stimulus-specific neural adaptation effects were evident within the first 10 s. Thus, our results confirm the utility of this EEG paradigm to assess stimulus-specific neural adaptation–a measure of neural tuning–in a rapid, reliable manner.

However, contrary to our hypotheses we did not find group differences among the neural adaptation effects in the ERP components we were able to examine. Because of the rapid presentation used in the paradigm, there are other ERP components typically associated with visual objects that we were not able to examine, and it is possible that groups might have shown differences on those components. For instance, we were only able to isolate two prominent ERP components consistent with early visual processing: the C1 and the P100. Prominent later components, such as the N100, P200, and N200, could not be resolved due to the overlap in neural responses to the stimuli during the stimulation blocks, or, if present, overlapped with the C1 and P100 response to the subsequent stimulus. The C1 and P100 are typically associated with processing of basic visual features (e.g., luminance, contrast) [46, 47], but they have also been shown to be sensitive to other factors including attention, and emotion [48], more so for the P100 than the C1. On the other hand, later ERP components, including the N100, P200 and N200, have been associated with object recognition and categorization [49–51]. Thus, these components may have been more sensitive to object-related processing in SZ and BD than the early components we were able to examine in our paradigm. It is possible, then, that the tuning effects seen for the P100 may be due to tuning of basic visual features rather than object-specific tuning of the stimulus itself. Future studies could adapt the current paradigm or use different, slower paradigms that would allow for resolution of these object-sensitive components.

Aside from this design limitation, there are other limitations present in the current study. Most participants were medicated; thus, we are unable to determine whether tuning deficits might be apparent in unmedicated people with SZ or BD. However, CPZ equivalents were not correlated with the difference scores, suggesting that antipsychotic medications did not influence our results. Another limitation was the use of only a few object exemplars. It may be possible that tuning deficits would be evident if stimulus-specific neural adaptation was assessed using a broader array of objects than those used in this study. Similarly, it is possible that tuning deficits might be more prominent for socially-relevant stimuli, in particular faces, given the well-documented deficits in social stimulus processing found in SZ [52–55]. The method used to display stimuli in the current study (i.e., transient onsets and offsets of stimuli) could also potentially be a limitation. Other methods, such as steady state visual paradigms (whereby the stimulus is periodically modulated in a sinusoidal-like fashion) or adaptation tasks (present two identical or different stimuli in succession and measures changes in neural activity) might have been better suited for examining tuning effects. These possible design choices can be explored in future studies to further examine if tuning deficits might be present in SZ or BD.

It remains unclear why there is an inconsistent pattern of results across the few studies that have directly assessed stimulus specific adaptation and neural tuning in these psychiatric conditions. The current results are consistent with a prior study from our laboratory, which also found no evidence of tuning abnormalities using multivoxel pattern analysis of fMRI data in people with SZ or BD [38], but they are inconsistent with two other studies that found effects consistent with tuning deficits in SZ [37, 56]. It is likely that several factors, such as the different neuroimaging modalities (EEG vs. fMRI), different paradigms (rapid serial visual presentation, multivariate pattern analysis/MVPA, fMRI adaptation), and even different stimuli, could be related to the inconsistent findings.

We also did not find any significant associations with symptom ratings, and it remains unclear why these associations were not found given that prior studies (e.g., [27]) have found associations with object processing. It is possible that, given the participants were not required to identify the objects, object processing was not fully engaged due to the task design. It is also possible that the lack of associations with symptoms was due to the fact that the patients in the current study were older patients with several years of clinical stability and most were medicated. Despite the lack of relationships to symptoms, exploration of neural tuning deficits in people with psychosis has only recently begun, and much work remains to be done to clarify whether or not abnormal visual tuning contributes to poor visual processing and, ultimately, poor daily functioning and increased symptomatology in people with SZ or BD.

In conclusion, the results of the current study showed the validity of the rapid serial visual presentation ERP method and complement findings from fMRI studies by examining adaptation and tuning effects at a different level of temporal resolution. However, they do not support our hypotheses related to reduced neural tuning in SZ or BD. Although the results do not show group differences, the findings have broader implications for assessment of visual processing in these disorders. Other types of visual stimuli for which psychiatric patients exhibit abnormal processing, such as faces, biological motion, and backward-masked stimuli, may be amenable to assessment using this type of stimulus-specific adaptation paradigm.
